# Effects of Greek Honey and Propolis on Oxidative Stress and Biochemical Parameters in Regular Blood Donors

**DOI:** 10.3390/jox12010002

**Published:** 2022-01-04

**Authors:** Ioannis Tsamesidis, Chinedu O. Egwu, Diana Samara, Dimitra Vogiatzi, Athanasios Lettas, Evgenia Lymperaki

**Affiliations:** 1 Department of Biomedical Sciences, International Hellenic University, 570 01 Thessaloniki, Greece; evlimper@gmail.com; 2 School of Dentistry, Faculty of Health Sciences, Aristotle University of Thessaloniki, 541 24 Thessaloniki, Greece; 3 Laboratory Medical Biochemistry, College of Medicine, Alex-Ekwueme Federal University Ndufu-Alike, Abakaliki 1010, Nigeria; echojay2010@yahoo.com; 4 Blood Bank Section, Naoussa General Hospital, 592 00 Naousa, Greece; dianasamara7@gmail.com (D.S.); athana.lettas@gmail.com (A.L.); 5 Department of Food Science and Nutrition, University of Aegean, 811 00 Mitilini, Greece; dimitravog1995@yahoo.gr

**Keywords:** Greek honey, Greek propolis, healthy blood donors, oxidative stress, biochemical parameters

## Abstract

**Background and objectives:** Honey products contain a lot of compounds, such as vitamins, enzymes, and minerals, which make honey and its products a great antioxidant with a critical role in health status. It is well accepted that honey and propolis can improve a lot of health problems when they are consumed in certain quantities. The objective of this study is to help regular blood donors improve their health status after donation. **Material and methods:** Eighty regular blood donor volunteers—30 males aged 19–61 and 30 females aged 21–64—were divided into 4 groups: group A (*n* = 20) consumed 2 spoons of Greek honey and 1 drop of propolis per day for 1 month, group B (*n* = 20) consumed 2 spoons of honey per day for 1 month, group C (*n* = 20) consumed 1 drop of propolis per day, and group D (*n* = 20) did not consume any Greek honey products. Blood samples were collected from all participants just before the consumption of the products, one month after the consumption, and six months after honey product consumption had ceased. All samples were analyzed for reactive oxygen species (ROS), lipid profiles, and ferritin levels. **Results:** The ROS were significantly (*p* < 0.05) lower in groups A, B, and C after the honey product consumption and increased significantly again after six months. No significant differences in lipid profiles were observed. Only triglyceride levels were increased after six months in all groups. On the other hand, ferritin levels were not statistically significantly decreased after six months in groups A and B, while they were increased in group C. **Conclusions:** In the present study, statistically significant decreases in ROS status was found after a small dose of honey product consumption, indicating a diet with an extra small dose of honey products after blood donation.

## 1. Introduction

Honey is a sweet flavored liquid that serves both as food and medicine and is produced by honey-bees secreting the nectars of flowers; honeydew honey (forest honey) is produced by the accumulation of plant-sucking insects [1,2]. It is well known that honey is composed of carbohydrates, water, essential oils, proteins, acids, minerals, and vitamins [3,4]. Moreover, it contains enzymes, such as catalase [3]. All antioxidants—endogenous or exogenous—can significantly reduce oxidation and protect the cell. Antioxidants, such as vitamins, enzymes, and other bioactive compounds (such as phenols and flavonoids), present in honey with antioxidant activity can neutralize ROS (Reactive oxygen species) production. The levels of enzymes (such as catalase) and vitamins (such as vitamin C and E) play a critical role in the antioxidant capacity of honey in health status. The percentage of these honey components determines the quality. Dzugan et al. demonstrated that the antioxidant activity of honey can be used as a biomarker to determine its variety [5].

Propolis of different classes contain resin, pollen, vitamins, flavonoids, and phenols. Flavonoids give the anti-inflammatory, anti-viral, anti-allergic, anti-cancer, anti-bacterial, and antioxidant effects of the compound [6,7]. Moreover, Pawel Olczyk and his team demonstrated that propolis could modulate fibronectin expression in the matrix of thermal injury.

On the other hand, oxidative stress plays an integral role in many degenerative diseases which could be neurological, cardiovascular, or metabolic in nature [8,9,10,11]. Among the neurodegenerative diseases are Alzheimer’s disease (AD), Parkinson’s disease (PD), and amyotrophic lateral sclerosis (ALS) [10,12], while cardiovascular diseases are represented by hypertension and myocardial infarction [9]. Among metabolic diseases, diabetes is the most common [11]. These diseases are characterized by extensive oxidative damage to lipids, proteins, DNA, and other important biomolecules, which destroys the endothelia and can lead to cell death if untreated.

Oxidative stress has also been known to aggravate hemoglobinopathies, such as sickle cell anemia, thalassemia, etc. Although oxidative stress is directly involved in the etiology of these diseases, oxidative damage to erythroid cells plays an important part in hemolysis [13]. These hemolytic diseases may affect the quality of blood and, hence, can influence blood donation by prospective donors. Checking the quality of blood and the health status of donors is a prerequisite before blood collection and for continued donation. Several antioxidants have been noted to have therapeutic potential against oxidative stress under hemolytic conditions. As our previous work [14] indicated, as regular blood donors’ oxidative stress is lower than among first-time blood donors, it will be helpful to introduce dietary supplements, such as honey and/or propolis, in order to modulate ROS production and, consequently, the oxidative status of the blood donors. Considering that honey and propolis have numerous health benefits, among which are wound healing, preventing acid reflux, reducing blood glucose levels, relieving signs of a cold, and several other benefits related to their antioxidant capacities and their ability to improve the lipid profile of subjects after at least of one month of consumption [15,16]. Moreover, honey has been reported to have antioxidant properties; hence, it can offer some therapeutic protection [17,18].

It is accepted that the chemical composition of honey—and, consequently, its bioactivity—depends on the geographical position and, consequently, on the botanical source of the harvested honey. Manuka honey, which originates from New Zealand, is very famous for its antibacterial activity in comparison with other types of honey around the world [19]. Stakos et al. demonstrated that some types of Greek honey originating from Mount Olympus have superior antibacterial and antioxidant properties [20]. Importantly, some of the honey types from Mount Olympus (North Greece) had higher antibacterial activity than Manuka honey.

Taking into consideration all these data and the benefits of honey, we elected to study the protective effect of Greek honey and Propolis from North Greece (Chalkidiki) against oxidative stress in regular blood donors. Moreover, the biochemical variations in blood cholesterol, HDL, LDL, triglycerides, and ferritin were performed too.

## 2. Materials and Methods

### 2.1. Study Design

**Figure 1:**
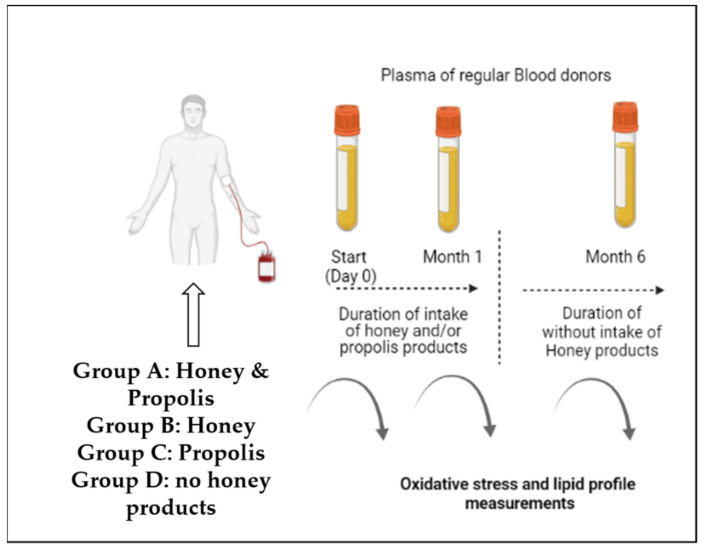
Flow chart of the experimental procedure.

A pilot study (Figure 1) was carried out from January 2020 to December 2020 during the COVID-19 pandemic at the Blood Bank of the Naousa Hospital in Greece. The blood donors (*n* = 60) were comprised of 30 males aged 19–61 years and 30 females aged 21–64 years. Daily consumption of Greek honey (2 spoons) and propolis (1 drop) was performed among individuals in group A, only honey was consumed by blood donors (BDs) in group B, and only propolis was consumed by group C in the same dose.

Each portion of the tested honey was fixed for the regular blood donors (rBDs) using special plastic spoons for honey intake provided to them with the honey. All participants (Group A–C) were instructed not to consume any other honey product during the course of the pilot study. The compliance of the volunteers was assessed by giving them a diary to record the dosage taken during the study. The research assistants followed up with the participants each week (4 times during 1 month) to ensure the ingestion as well as the correct dosage.

All participants provided written informed consent before the study and were asked to fill out a short questionnaire about sociodemographic characteristics, such as age, sex, body mass index (BMI), smoking habits, and the residence of blood donors. All volunteer rBDs were selected based on their health status and excluding drug and supplement intake. It was suggested to participants that they not change their dietary habits during the research study. Blood samples were collected from all participants just before blood donation (day 0), after 1 month of honey products consumption (1 month), and 6 months after honey product consumption had ceased. All samples were analyzed for their lipid profiles and ferritin levels and were stored at −80 °C to measure ROS levels.

### 2.2. Honey Product Samples

The Greek honey products (blossom honey and propolis), produced in Chalkidiki, Greece, were gifted from the *Sithon (Σιθων) Honey Company* (http://www.honeysithon.gr/en/ accessed on 5 April 2021). The botanic source of the honey was polyfloral, and the location of the harvesting was in Chalkidiki.

### 2.3. Ethical Statement

Healthy regular blood donors, all adults, provided written, informed consent before entering the study. The study was conducted in accordance with Good Clinical Practice guidelines and the Declaration of Helsinki. Ethical approval to perform the present study was obtained from the Ethical Committee of the General Hospital of Naousa, Greece. The confidentiality of participants was wholly preserved.

### 2.4. Analysis of ROS Values

The analysis of reactive oxygen species was performed using the cell-permeable, ROS-sensitive probe 2′,7′-dichlorodihydrofluorescein diacetate (H_2_DCFDA) fluorescing at 520 nm (λex 480 nm) upon oxidation. An H_2_DCFDA probe (0.5 mM stock solution in DMSO) (incubated for 30 min) was incubated in the human plasma containing the plasma membrane-vesicles (PMVs) responsible for the ROS presence in human plasma, as previously described [21]. The analysis of the ROS was performed at all tested time points (before and after the administration of the honey products). The monitoring of the measurement of the fluorescence of the desired suspensions in 96-well black microplates was performed using a *Tecan* fluorometer.

### 2.5. Analysis of Blood Lipids and Ferritin

All biochemical parameters, total cholesterol, HDL, LDL, triglycerides were measured using Cobas Integra 400 Plus and ferritin with Cobs e411Elecsys automated analyzers.

### 2.6. Statistical Analysis

To compare the ROS and biochemical markers of the different groups at different time points, SPSS tool version 22.0 was utilized. Descriptive statistics, presented as means ± standard deviations, were performed. Additionally, an inferential statistical analysis (t-test) was used to investigate the possible differences between the two blood groups regarding biochemical markers’ statuses. A one-way ANOVA test further evaluated possible differences in the lipidemic and anemia predisposition among blood groups. In all statistical analyses, the level of significance (*p*-value) was set at α = 0.05.

## 3. Results

The average ROS levels and biochemical parameters are presented in Table 1.

**Figure 2:**
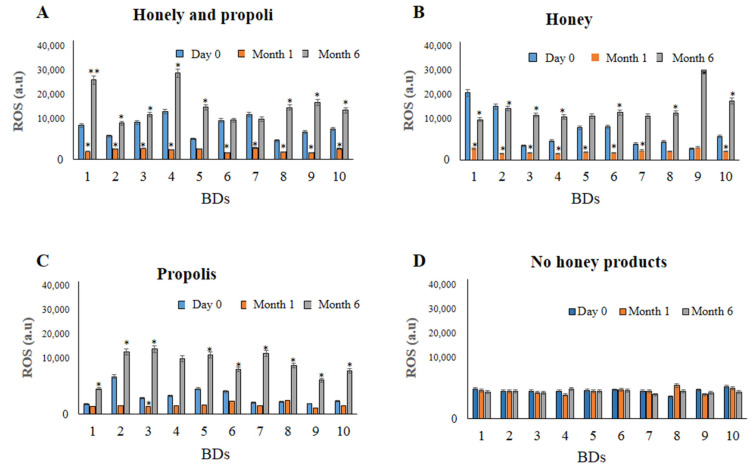
ROS levels (a.u) of selected blood donors (*n* = 10). (**A**) Group A, Greek honey and propolis; (**B**) group B, honey;(**C**) group C, propolis; and (**D**) group D; no honey products intake Data are the averages ± SDs of the 5 measurements. Significant differences from day 0 are represented as * *p* < 0.05 and ** *p* < 0.001.

Table 2 presents all the biochemical parameters and ROS levels of the regular blood donors before and after honey and/or propolis consumption. In details, total cholesterol levels appeared to be the same or lower in BDs following honey product intake at all of the tested time points without any statistically significant difference. Further, no statistically significant differences were observed between the three groups regarding HDL levels; however, these levels appeared to be influenced more than the cholesterol levels. All of the honey products appeared to slightly decrease the HDL levels—especially the combination of propolis with blossom honey. On the other hand, triglycerides levels were increased after the intake of honey and propolis in all three groups, indicating a statistically significant difference between day 0 and month 6 (*p* < 0.05). When comparing the ferritin levels of BDs at day 0 with those at month 6, a statistically insignificant decrease was observed among groups A and B, while a statistically significant increase was revealed for group C (*p* < 0.05). Healthy volunteer blood donors (BDs) undergoing blossom honey intake for a one-month period presented a statistically significant reduction in the ROS levels in comparison with their initial amounts (*p* < 0.001). In Figure 2, the corresponding ROS levels of the individuals are presented at the selected time points (i.e., the ROS levels of selected blood donors). Interestingly, after the first month of honey use, as well as 6 months later, the ROS levels returned to normal or increased for all BDs (Figure 2B). The same trend of the reduction and consequent increase in ROS levels was also observed for the BDs consuming propolis alone as well as among those consuming propolis in combination with blossom honey (Figure 2A,C). No statistically significant differences were observed for BDs without the intake of honey products (Figure 2D).

## 4. Discussion

Honey has been used since ancient times as a remedy for various diseases, such as wounds, diseases of the gut, sore throat, and earaches, and has antimicrobial, anti-inflammatory, and anticancer properties. The antioxidant activity of honey is measured using different analytical methods, and the antioxidant components of honey and its products (such as propolis) can contribute to serum antioxidant activity [22,23,24,25]. The question is the dose of honey and propolis consumption and the duration of their benefits. Our study revealed that after one month of consumption of two spoonfuls per day of honey and/or one drop of propolis alone or in combination can influence not only the oxidative status of healthy blood donors but also triglycerides and ferritin levels in long term. This phenomenon was noticed after the repeated biochemical examinations six months after the last consumption of the products. According to the literature, the health benefits of honey and propolis are due to phenols, chrysin, quercetin, caffeic acid, galangin, acacetin, and pinocembrinare, which are responsible for the molecular mechanisms which activate the mitochondrial pathway, modulate oxidative stress, and reduce the inflammation inhibiting angiogenesis [7,26]. Our study is in accordance with other reports regarding the synergistic effect of honey and propolis that indicate therapeutic, antibacterial, and antioxidant properties, as well as the potential to effectively influence the oxidative status, thus reducing the ROS and consequently improving the health status conditions. The elevated ROS production observed in our study after five months without any honey intake could be attributed to some cellular processes of upregulation due to an imbalance that was created after the sudden removal of honey product intake. A permanent, balanced diet of antioxidants appears to be the ideal solution for balanced ROS levels.

On the other hand, honey and propolis consumption alone or in combination showed no significant differences in lipid profiles. This may be due to the small dose of the extra product for a short period of time, as previously reported [27]. Another reason is that lipid profile changes depend on the type of honey, as was described by Mohammadimanesh and his team [28]. All tested groups, as expected [27], presented moderately elevated triglyceride levels without exceeding normal values after one month of honey product consumption. Specifically for triglycerides, groups undergoing propolis intake alone or in combination with honey presented a further increase after long-term observation in comparison with the group undergoing honey intake only. It is worth noting that honey alone or in combination caused a slight reduction in LDL and total cholesterol levels, possibly because of its composition in bioactive substances [26]. For the first time in the literature, we observed the modification of the biochemical parameters after one month of consumption and six months of observation, while Noori S. et al. [29] monitored biochemical parameter modifications during a two-week control period. Blood ferritin levels indicate iron sores and high ferritin levels indicate an iron storage disorder or an inflammation. In our study, we observed low ferritin levels where only after propolis intake was an increase noticed. This finding suggests a transitory influence of propolis ingestion on iron absorption and shows no negative effect under the described experimental conditions. Our study indicates that there might be some differences between the short-term and long-term effects of propolis supplementation. To gain a complete picture of propolis alone and in combination with honey treatment, it would be necessary to conduct a randomized clinical trial with a larger number of participants and different types of honey and propolis.

## 5. Conclusions

In conclusion, our data suggest the controllable dietary intake of Greek honey and/or Greek propolis for one month after blood donation. Their beneficial role is presented due to their known components’ abilities to reduce reactive oxygen species after short-term use and induce a non-pathological increase in ferritin levels. On the other hand, avoiding honey product intake led to a further increase in ROS levels; for this reason, we propose the use of honey products at specific dosages for more than one month. Their role is differentially related to conditions and doses and can be used as an alternative drug to stimulate the immune system [30] and modulate oxidative stress in pathological conditions.

## Figures and Tables

**Table 1:** Characteristic of all study participants.

Blood Groups (*n* = 80)				
	A (*n* = 20) Mean (±SD)	B (*n* = 20) Mean (±SD)	C (*n* = 20) Mean (±SD)	D (*n* = 20) Mean (±SD)
**Sex, Male (%)**	50	50	50	50
**Age**	44 (±11.35)	45 (±7.52)	47 (±7.31)	42 (±3.17)
**BMI**	24.8 (±2.11)	24.3 (±1.65)	23.4 (±1.94)	24.2 (±1.41)

**Table 2:** Biochemical parameters and ROS levels of the regular blood donors before and after honey and/or propolis consumption. Data are the averages ± SDs of the 3 measurements. Significant differences from day 0 are represented as * *p* < 0.05 and ** *p* < 0.001.

	Group A *(Honey and Propolis)*			Group B *(Honey)*			Group C *(Propolis)*			Group D (No Honey Products)		
Biochemical Parameters	Day 0	Month 1	Month 6	Day 0	Month 1	Month 6	Day 0	Month 1	Month 6	Day 0	Month 1	Month 6
**Total** **Cholesterol (mg/dL)**	185.5 ± 25	185.3 ± 21	176.2 ± 19	212.1 ± 17	215.5 ± 18	193.5 ± 16	176 ± 14	182.4 ± 15	181.4 ± 19	181.2 ± 21	184.3 ± 15	183.5 ± 19
**HDL (mg/dL)**	47.1 ± 6	45.1 ± 5	39.6 ± 4	45.5 ± 7	44.2 ± 3	43.2 ± 6	48.6 ± 4	52 ± 5	47.5 ± 3	43.2 ± 4	45.5 ± 6	46.2 ± 7
**LDL (mg/dL)**	114.7 ± 15	116.1 ± 14	104.1 ± 16	137.8 ± 18	136.2 ± 13	116.7 ± 19	118.3 ± 15	111.2 ± 18	108.2 ± 11	125.5 ± 18	130.4 ± 17	129.2 ± 21
**Trig. (mg/dL)**	118.7 ± 11	122.3 ± 16	163.7 * ± 15	144 ± 12	157.7 ± 16	167.4 * ± 12	92.8 ± 9	90.4 ± 15	145.5 * ± 12	114.2 ± 15	123.5 ± 11	121.4 ± 17
**Ferritin (ng/mL)**	53.8 ± 28	62.5 ± 23	38.3 ± 18	96.9 ± 35	114.6 ± 45	65.4 ± 35	67.7 ± 28	68.7 ± 23	101.3 * ± 32	62.1 ± 25	73.5 ± 29	67.5 ± 18
**ROS** **(a.u)**	10,880 ± 254	3159 * ± 352	18,024 ** ± 340	7854 ± 158	2760 * ± 325	19,863 * ± 124	5374 ± 305	2793 * ± 205	15,013 * ± 167	7856 ± 158	8647 ± 114	8025 ± 201

**Abbreviations: HDL**, high-density lipoprotein; **LDL**, low-density lipoprotein; **Trig**., triglycerides; **ROS**, reactive oxygen species.

## Data Availability

Data is contained within the article.
